# Remembering John Steele and his models for understanding the structure and function of marine ecosystems

**DOI:** 10.1093/plankt/fbz042

**Published:** 2019-10-03

**Authors:** Thomas R Anderson, Wendy C Gentleman

**Affiliations:** 1 NATIONAL OCEANOGRAPHY CENTRE, EUROPEAN WAY, SOUTHAMPTON SO14 3ZH, UK; 2 Department of Engineering Mathematics, Dalhousie University, 5217 Morris St, PO Box 15000, Halifax, NS B3J 1B6 Canada

**Keywords:** marine ecosystem modelling, plankton dynamics, zooplankton mortality, functional response

## Abstract

John Steele (1926–2013) is remembered for his ecosystem modelling studies on the role of biological interactions and environment on the structure and function of marine ecosystems, including consequences for fish production and fisheries management. Here, we provide a scientific tribute to Steele focusing on, by means of example, his modelling of plankton predation [Steele and Henderson (1992) The role of predation in plankton models. *J. Plankton Res.*, **14**, 157–172] that showed that differences in ecosystem dynamics between the subarctic Pacific and North Atlantic oceans can be explained solely on the basis of zooplankton mortality. The study highlights Steele’s artistry in simplifying the system to a tractable minimal model while paying great attention to the precise functional forms used to parameterize mortality, grazing and other biological processes. The success of this and other works by Steele was in large part due to his effective communication with the rest of the scientific community (especially non-modellers) resulting from his enthusiasm, use of an experiment-like (hypothesis driven) approach to applying his models and by describing simplifications and assumptions in scrupulous detail. We also intend our contribution to remember Steele as the consummate gentleman, notably his humble, behind-the-scenes attitude, his humour and his dedication to enhancing the careers of others.

## INTRODUCTION

**Fig. 1:**
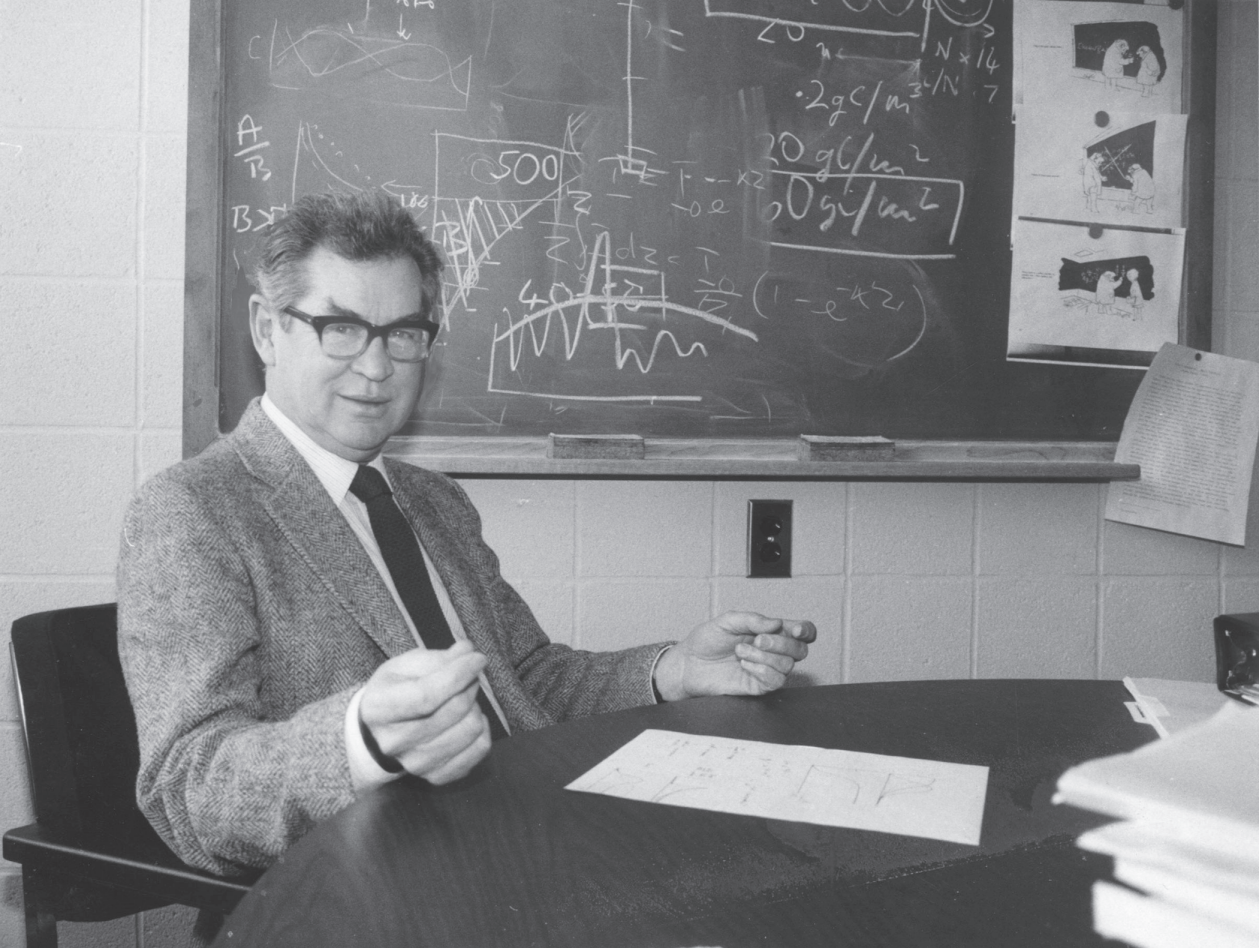
John Steele having fun with mathematics (1984). © Getty Images

Understanding the dynamics of marine ecosystems and associated biogeochemistry is a challenging task given the complexity of ecosystems and associated interactions between organisms and their physico-chemical environment. For the two of us, John Steele (1926–2013; Fig. 1) should be remembered and revered when it comes to demonstrating the use of ecological modelling as a research tool to understand these complexities, both in his methodology and approach, and in the way he reached out to the scientific community as a whole with his ideas and findings.

Born in Scotland, Steele was educated at George Watson’s College in Edinburgh and subsequently received a first class degree in mathematics at University College, London in 1946. His first job was with the Royal Air Force in Farnborough, developing numerical methods to calculate the optimal trajectories for ground-to-air missiles. Leisure time at weekends was spent sailing off the coast near Southampton, and it was this “desire to spend time messing about in boats” (Steele, 1997) that led him to a new passion: the sea. Travelling back north, Steele took up a position working on fisheries management at the Marine Laboratory in Aberdeen, where he would remain from 1951 to 1977. His early work was decades ahead of its time, involving a detailed consideration of the relationship between the annual cycle of production and the physical environment, including rates of mixing, the vertical structure of the water column and the three-dimensional circulation (Steele, 1956, 1958, 1959). The link between environment, food web structure and higher trophic levels was an ongoing theme throughout his career, in which he cultivated many interests including plankton community structure, ecosystem stability, physical–biological coupling, patchiness, controls on phytoplankton production, closure, prey switching, trophic cascades, individual-based and end-to-end models. He made major contributions to fisheries science, emphasizing the link between fish and the lower-trophic–level ecosystem, as well as the interdisciplinary nature of understanding the relationship between environment and biological resources (e.g. Steele, 1996; Steele, 2012; Steele *et al.*, 2019). A defining moment was the publication of his book, *The Structure of Marine Ecosystems* (Steele, 1974), which was reviewed by Sir Robert May who describes Steele as a “skilled helmsman” who could “steer between the Scylla of a multiparameter computerized Goon Show and the Charybdis of total abstraction” (May, 1974). Heady praise indeed!

Steele’s achievements were stellar throughout his career, not only in scientific publication but also through his enthusiasm for oceanography and the many people’s lives he influenced, his service as director of the Woods Hole Oceanographic Institution 1977–1989 and as recognized in many honours including the Alexander Agassiz Medal from the U.S. National Academy of Sciences in 1973, election to the Royal Society of Edinburgh in 1968, the Royal Society in 1978 and as Fellow of the American Academy of Arts and Sciences in 1980. Curiosity was his “primary excitement” and so he was “engaged in everything” (H. Ducklow, quoted in Cook, 2013). Our main aim here is to showcase the strengths of Steele’s approach to science, which must surely inspire the next generation of researchers. By means of example, we reconstruct and analyse Steele’s exemplary model of the contrasting plankton dynamics in the North Atlantic and subarctic Pacific oceans (Steele and Henderson, 1992; hereafter SH92). We also intend our contribution to serve as a tribute to John, remembering not only his immense scientific prowess but also his humble, behind-the-scenes attitude, his humour and his dedication to enhancing the careers of others.

## THE STEELE AND HENDERSON (1992) MODEL

**Fig. 2:**
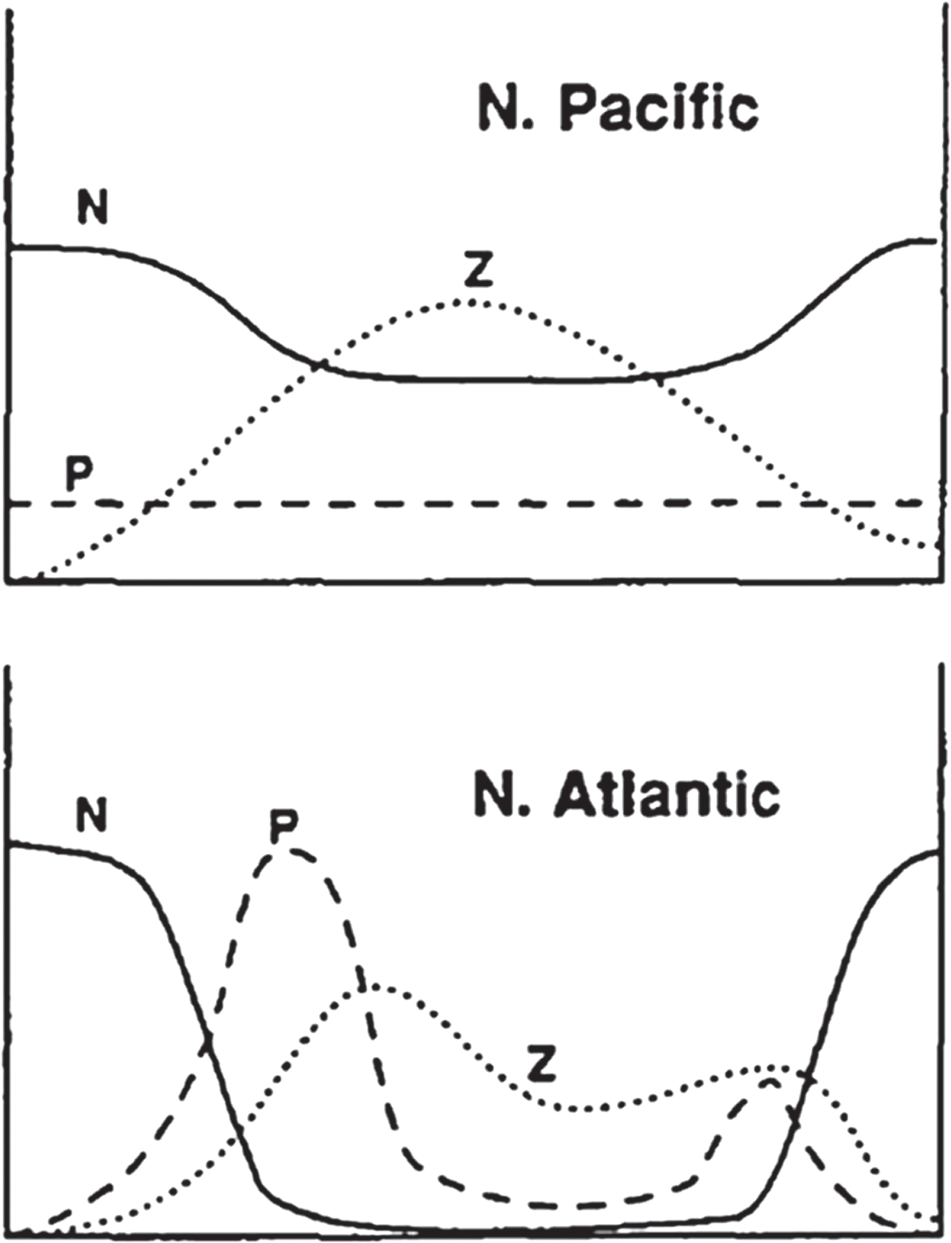
Schematic presentation of the annual cycles associated with the North Pacific and Atlantic Oceans (SH92).

The SH92 model is a seminal work that has had a long-standing impact through its emphasis on understanding the importance of the precise functional forms of grazing and mortality in the functioning of marine ecosystems, both in terms of interpreting observational data (Ohman and Hirche, 2001; Gentleman *et al.*, 2012) and from a modelling perspective (Edwards and Brindley, 1999; Edwards and Yool, 2000; Fulton *et al.*, 2003; Anderson *et al.*, 2010). Steele demonstrated his approach by using it to examine the differences in the seasonal cycles of phytoplankton and nutrients in the contrasting North Atlantic and subarctic Pacific oceans. The former has a pronounced spring bloom and levels of nitrate decline over summer to levels that may limit phytoplankton, whereas the subarctic Pacific is a high-nutrient low-chlorophyll (HNLC) system where phytoplankton concentrations remain low, and nutrient concentrations high, throughout the year (Fig. 2). Steele noted that there are also important differences in species composition and size structure between the two ecosystems (Parsons and Lalli, 1988), with HNLC dominated by small phytoplankton and microzooplankton, whereas the North Atlantic food web is characterized by diatoms and mesozooplankton during the spring outburst (SH92). Various hypotheses had been proposed to account for these differences in ecosystem structure, notably bottom-up limitation by iron (Martin and Fitzwater, 1988) versus top-down control by grazing (Frost, 1987). Steele (with Eric Henderson) set about constructing a simple model (SH92) to undertake his own analysis. The ecosystem structure and environmental forcing were kept remarkably simple, whereas maximum attention was paid to the precise way in which plankton groups interact, especially the functional forms of grazing and zooplankton mortality. Note that functional response and model closure had previously been examined in Steele and Henderson (1981). The model of SH92 extended the work by representing dynamic nutrients, including physical exchanges with deep water.

**Fig. 3:**
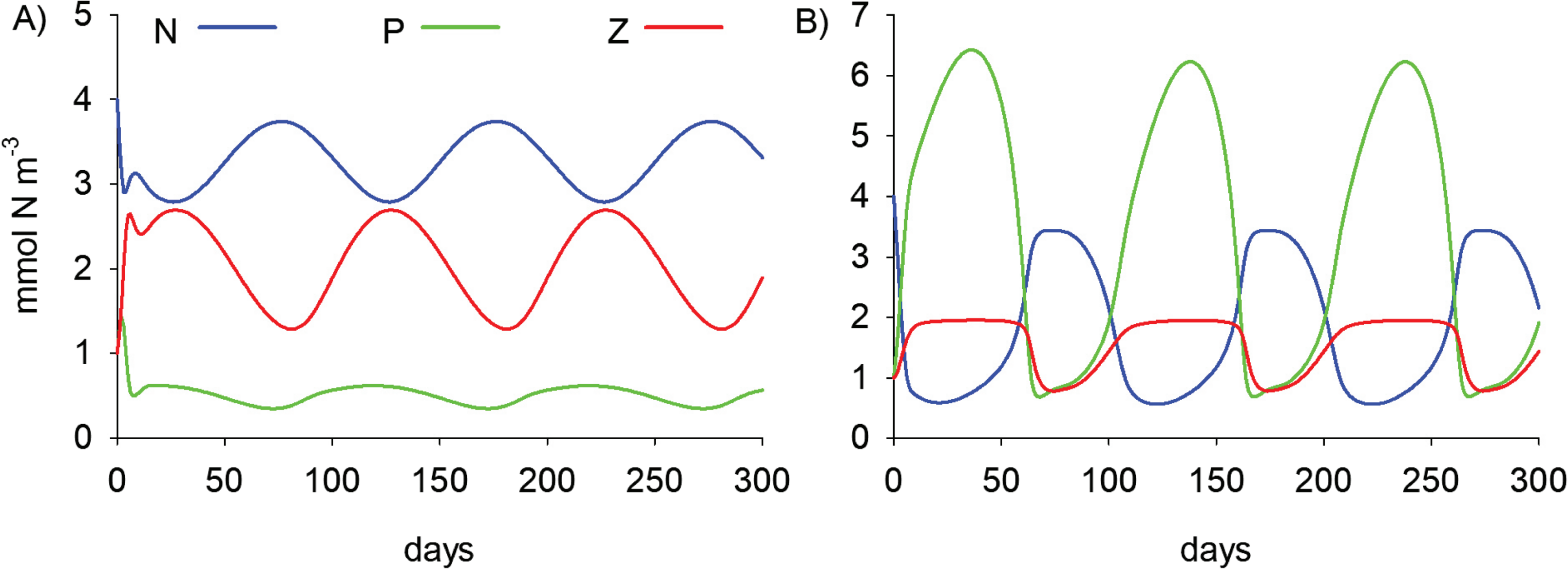
Model sensitivity to parameter m_Z_ (zooplankton mortality), giving rise to (**A**) subarctic Pacific (m_Z_ = 0.1 d^−1^ (mmol N m^−3^)^−1^) and (**B**) North Atlantic (m_Z_ = 0.5 d^−1^ (mmol N m^−3^)^−1^) dynamics. Results correspond to those shown in Fig. 6A and B of SH92.

### Model description

The SH92 model has three state variables, phytoplankton (P), zooplankton (Z) and nitrate (N), with rates of change described by the following differential equations that represent a “minimal set of interactions” (SH92):

(1) \begin{equation*} \frac{dP}{dt}={\mu}_PP-f(P)Z-{\Gamma}_P{k}_{mix}P \end{equation*}

(2) \begin{equation*} \frac{dZ}{dt}=\beta f(P)Z-\beta {m}_Z{Z}^w-{\Gamma}_Z{k}_{mix}Z\end{equation*}

(3) \begin{equation*} \frac{dN}{dt}=-{\mu}_PP+\left(1-\beta \right)f(P)Z+{k}_{mix}\left({N}_{deep}-N\right). \end{equation*}

Note that we are using parameter symbols in line with modern marine ecosystem models, rather than those used by SH2. The terms in the phytoplankton equation are growth, grazing and mixing. Specific growth rate, μ_P_, is given by

(4) \begin{equation*} {\mu}_P={\mu}_P^{max}\left(1-\frac{P}{\gamma}\right)\left(\frac{N}{k_N+N}\right)I(t), \end{equation*}

where ${\mu}_P^{max}$ is maximum phytoplankton growth rate (d^−1^), parameter γ is carrying capacity (mmol N m^−3^) and k_N_ is the half saturation constant for nutrient uptake. Time-varying irradiance, I(t), is specified as a sine curve:

(5) \begin{equation*} I(t)=1+a.\mathit{\sin}\left(\frac{2\pi t}{\Omega}\right), \end{equation*}

where *a* is amplitude and Ω is periodicity (days). Zooplankton functional response is

(6) \begin{equation*} f(P)=\frac{gP^n}{k_g+{P}^n}. \end{equation*}

The functional response has parameters g, the maximum grazing rate (d^−1^), k_g_, the half saturation constant and the shape parameter, n. Setting *n* = 1 gives a hyperbolic Type II functional response, whereas *n* = 2 is an S-shaped Type III response (Gentleman and Neuheimer, 2008).

The zooplankton equation has terms for growth, mortality and mixing. Growth is the product of grazing and an efficiency coefficient, β. Mortality is either linear (w = 1; m_Z_ then has units d^−1^) or quadratic (w = 2; m_Z_ units d^−1^ (mmol N m^−3^)^−1^). The nitrate equation has terms for phytoplankton uptake, nutrient regeneration and mixing. Steele assumed that nitrate in the surface layer mixes (parameter k_mix_) with a deep high-nutrient source with concentration N_deep_. Phytoplankton and zooplankton can potentially avoid mixing losses through morphological and behavioural means. We include this in the model as parameters Γ_P_ and Γ_Z_ (mixing occurs when Γ_P_ or Γ_Z_ = 1 but not when these parameters are set to zero). Concentrations of P and Z are assumed to be zero in the bottom layer. The model is coded in R using the EMPOWER framework (Anderson *et al.*, 2015) and is available on request to the first author.

**Fig. 4:**
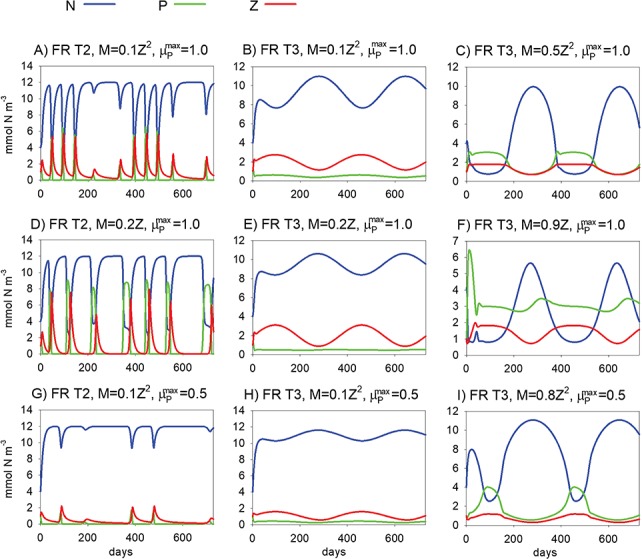
Model simulations examining sensitivity to functional response (FR, Type 2 or 3), the functional form of mortality (M: Linear and Quadratic), mortality rate (parameter m_Z_) and maximum phytoplankton growth rate $\Big({\mu}_P^{max}\Big)$. Base parameter values as for the run shown in Fig. 3, except Γ_P_ = 1, N_deep_ = 12 mmol N m^−3^ and Ω = 365 days.

## RESULTS

The results in SH92 demonstrate how differences in ecosystem dynamics between HNLC systems (the North Pacific) and seasonal high-latitude systems (the North Atlantic) can be generated solely on the basis of zooplankton mortality, parameter m_Z_. We start by reproducing this finding (SH92 Fig. 6) in which grazing is specified using a type III functional response (*n* = 2) with g = 1.0 d^−1^ and k_g_ = 1.0 (mmol N m^−3^)^2^ and quadratic zooplankton mortality (w = 2) with m_Z_ = 0.1 (Fig. 3A) and 0.5 d^−1^ (mmol N m^−3^)^−1^ (Fig. 3B). Nitrate is subject to mixing (k_mix_ = 0.3 d^−1^; N_deep_ = 4.0 mmol N m^−3^), whereas phytoplankton and zooplankton are not (Γ_P_ = Γ_Z_ = 0). Remaining parameters are also assigned values as in SH92: ${\mu}_P^{max}$ = 1.0 d^−1^, γ = 10 mmol N m^−3^, k_N_ = 0.5 mmol N m^−3^, β = 0.5, *a* = 0.5 and Ω = 100 days. Results show that low mortality (m_Z_ = 0.1) allows zooplankton to thrive and exert strong top down control of phytoplankton biomass, leading to the HNLC state (Fig. 3A). In contrast, increasing m_Z_ to 0.5 gives rise to a North Atlantic situation where P is seasonally high relative to N and Z. Note that the predicted ecosystem dynamics are stable in both cases, showing only slow changes in parallel with the 100-day simulated irradiance cycle.

It is not only magnitude that is important but also the precise functional forms of equations for mortality and grazing (Steele, 1974; Steele and Henderson, 1981). We now focus on functional forms, recreating the analysis of SH92 but extending the work to examine the effect of bottom-up control on phytoplankton via iron (focusing on parameter ${\mu}_P^{max}$). Minor improvements were made to the realism of the simulations, of the kind that would likely be approved by Steele, if he were still with us today. His later works show phytoplankton contributing to export (Steele, 2009; Steele and Gifford, 2010; Steele, 2012), and we thereby assumed that they are passive and subject to mixing (Γ_P_ = 1), using k_mix_ = 0.1 (this value was used in many of the simulations in SH92, albeit for nutrients). Deep nutrient, N_deep_, is assigned a value of 12 mmol m^−3^, which is representative of nitrate concentrations in the subarctic Pacific and North Atlantic oceans (e.g. Anderson *et al.*, 2015). We also extended the periodicity of irradiance, Ω, to a full annual cycle, 365 days. The results of Fig. 3A and B are qualitatively reproduced in Fig. 4B and C with these new parameter settings. Crucially, it is the qualitative differences that were of primary importance to Steele when seeking to understand the contrasting ecosystem dynamics of the two oceans.

Re-running the low m_Z_ simulation (Fig. 4B) with a Type II functional response leads to instability manifest as oscillatory behaviour, where the otherwise HNLC state is punctuated with frequent short-lived blooms (Fig. 4A). Predicted phytoplankton concentrations go extremely low (<10^−7^ mmol N m^−3^), highlighting the instabilities that occur at low prey density (potential extinction) because clearance rates increase as prey density decreases when using a Type II functional response (Steele and Henderson, 1981; Gentleman and Neuheimer, 2008).

The simulations in Fig. 4A–C are next repeated, except using linear instead of quadratic mortality (Fig. 4D–F). The predicted oscillations become even more pronounced when a Type II functional response is combined with linear mortality as phytoplankton concentrations decline to below 10^−21^ mmol N m^−3^ (Fig. 4D). The use of linear mortality generally promotes instability (Steele and Henderson, 1981). Stability is restored, giving rise to an HNLC state, when the Type II response is replaced by Type III (Fig. 4E), emphasizing the importance of functional response in stabilizing ecosystem dynamics. Sensitivity to functional response was likewise noted by Franks *et al.* (1986) when examining solutions of a plankton model similar to SH92. Increasing mortality to 0.9 d^−1^ brings about a rather strange state in which P and Z are relatively constant, with strong seasonal variation in nutrients (Fig. 4F).

The iron hypothesis (Martin and Fitzwater, 1988), namely that iron limits growth and, via bottom up control, phytoplankton biomass, came to the fore in the years following the publication of SH92, especially with stimulation of blooms during *in situ* iron addition experiments (e.g. Coale *et al.*, 1996; Boyd *et al.*, 2000). The role of iron as a controlling factor in marine ecosystems remains topical today (e.g. Person *et al.*, 2018; Schulz *et al.*, 2018). As an interesting extension to the work of SH92, we therefore use the model to examine the role of iron by decreasing maximum phytoplankton growth rate, ${\mu}_P^{max}$, which acts as proxy for iron limitation (Anderson *et al.*, 2015), by 50%. Returning mortality to quadratic, the resulting impact on the predicted seasonal cycles of P, Z and N is shown in Fig. 4G–I. These results are remarkably similar to those in Fig. 4A–C, albeit with a higher mortality rate in Panel I, illustrating that inclusion of iron in the analysis does not alter the fundamental conclusions reached in SH92.

## THE ARTISTRY OF SH92

The word “artistry”, meaning creative skill or ability, might seem an unusual descriptor for a modelling study, but it is entirely appropriate in the case of SH92. The work is a masterpiece that illustrates how modelling is as much an art as it is a science. Just what should and should not be included in models is the subject of contentious debate (e.g. Anderson, 2005, 2006, Le Quéré, 2006), and there are various other murky worlds including tuning parameters, quantifying uncertainty, validation, etc. Steele was a master when it came to mathematically abstracting the world into models, and complexity was never added for its own sake. When referring to his paintings of sunflowers, Van Gogh once said “Instead of trying to render exactly what I have before my eyes, I use colour more arbitrarily to in order to express myself forcefully” (Bailey, 2013). The word “arbitrarily” might somehow imply a casual approach, which was of course not the case. When it came to modelling, Steele was the Van Gogh of his era, expertly selecting his subject matter. “To set up a mathematical model of the process of production”, he wrote, “it is necessary to put some of these loosely defined relations into a rigid structure and to neglect the others completely. Such a procedure appears arbitrary, but it is only in this way that the logical consequences of these relations can be explored” (Steele, 1958).

The SH92 study shows how a single factor, zooplankton mortality, can explain the difference between ecosystem dynamics in the North Atlantic and subarctic Pacific oceans. We believe that Steele’s genius in this regard derives, at least in part, from his experience of mathematics, which gave him a unique perspective regarding systems dynamics and sage awareness of cause and effect. Breadth of knowledge also helped him in formulating the most appropriate hypotheses. He emphasized, for example, the importance of the spatio-temporal scales of interaction between ecosystems and environmental forcing, contrasting terrestrial and aquatic systems (Steele, 1991; Steele and Henderson, 1994; Steele *et al.*, 2019). Intrinsic properties of ecosystems were likewise of great interest. The last time that I (T.R.A.) saw John was at a EURO-BASIN synthesis workshop in Lisbon in November 2012. I was at that time doing some work comparing terrestrial and marine systems, and the two of us discussed the “green world hypothesis” that proposes that primary producers flourish in conditions where the action of herbivore populations on plants is restricted by predators and parasites (Hairston *et al.*, 1960). Put simply, the level of predators forces other system components to relatively high or low concentrations (Steele and Henderson, 1981). Thus, high numbers of predators driven by low mortality should keep phytoplankton biomass in check and lead to the HNLC state and vice versa for the North Atlantic ecosystem. It seems so obvious and simple, yet it takes great vision to be able to see what to focus on among the myriad of processes and interactions within ecosystems.

While major simplifications were made in terms of model structure, Steele’s work in SH92 and many other publications demonstrates the importance of paying close attention to the precise functional forms used to parameterize mortality, grazing and other biological processes. More recently, Flynn (2005) likened not doing so as being equivalent to building castles on sand. Yet dysfunctionality, i.e. behaviour contrary to that expected from observation and experiment, can all too easily creep into model equations for various reasons including ignorance, indifference and inertia (Anderson and Mitra, 2010). It is perhaps remarkable that modellers are, in some cases, enthusiastic about increasing complexity by adding state variables and/or processes in their models but without, for example, undertaking detailed sensitivity analysis of the parameterizations involved. Few studies of parameter sensitivity have been carried out in global ocean models but those that have showed marked sensitivity to, for example, the precise functional forms for grazing and trophic transfer (Anderson *et al.*, 2010, 2013). What happened to the old art of rigorously testing model parameterizations in simple physical frameworks such as slab models? Mike Fasham, for example, carried out and documented 201 runs of his well-known marine ecosystem model (Fasham *et al.*, 1990), prior to it being published (Anderson and Bryden, 2015). Sensitivity to model parameterizations may be a common feature of biological models (Wood and Thomas, 1999; Fussmann and Blasius, 2005; Sailley *et al.*, 2013), and for multiple interacting state variables in an ecosystem, the implication is therefore that realism and precise formulation are required in these parameterizations, and moreover, the associated representation of the physico-chemical environment (Anderson, 2005).

The SH92 paper is the classic citation when it comes to justifying the functional form of closure in models (e.g. recent examples include Rowe *et al.*, 2017; Chen and Smith, 2018). It also paved the way for further studies on the factors controlling HNLC and other systems (e.g. Fasham 1995; Chai *et al.* 1996; Pitchford and Brindley, 1999; Strom *et al.*, 2000). We extended the SH92 analysis to look at the role of iron as a bottom-up control of HNLC. Decreasing phytoplankton growth rate (a proxy for iron limitation) makes it easier for zooplankton to prevent phytoplankton blooming, in turn meaning that zooplankton mortality rate can be higher (giving rise to a smaller zooplankton population) while still maintaining the HNLC state. Our results nevertheless show that even with the slower algal growth rate, differences in ecosystem dynamics between the North Atlantic and subarctic Pacific oceans can be explained solely on the basis of differences in the rate of zooplankton mortality.

**Fig. 5:**
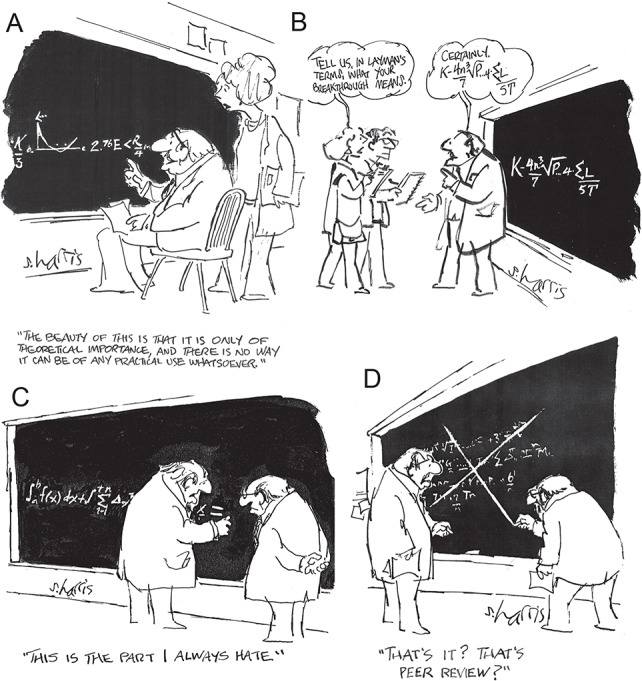
Sidney Harris cartoons: (**A**) The beauty of this is that it is only of theoretical importance …; (**B**) Tell us, in layman’s terms, what your breakthrough means; (**C**) This is the part I always hate; (**D**) That’s it? That’s peer review? © Sidney Harris.

## COMPLETE SCIENTIST AND CONSUMMATE GENTLEMAN

Steele was the complete scientist. For example, Bob Gagosian remarked of him, “It’s the people who go to sea, who develop the theories, who write the computer code, who construct and use the models, who do the analyses, who interpret the results, and make the conclusions that advance the field” (Gagosian, 2014). In this section, we focus on two of Steele’s greatest strengths that contributed to this completeness, namely his effective communication with the rest of the scientific community by making his math meaningful and engaging and the way he went about using models to test hypotheses. Before doing so we note that, as with most great scientists, Steele was self-critical and fully aware of the pros and cons of scientific method, especially with regard to modelling. He had a wry sense of humour and could have a quip, at least privately with himself, about maths for maths sake and the danger of mathematicians and modellers losing sight of the real world and becoming ensconced within a world of equations that have little more meaning than abstract theory. We know that he enjoyed the cartoons of Sidney Harris (e.g. Harris, 1990) and, by means of introduction to this section, have selected four examples that he may have particularly liked (Fig. 5; note that cartoons C and D appear pinned to the blackboard in the photo shown in Fig. 1). The cartoons are the antithesis of Steele: (i) practical application was of primary importance; (ii) Steele was good at communicating the science he did to biologists; (iii) he enjoyed math and the challenge of modelling; (iv) he was always conscientious and constructive in his criticism.

**Fig. 6:**
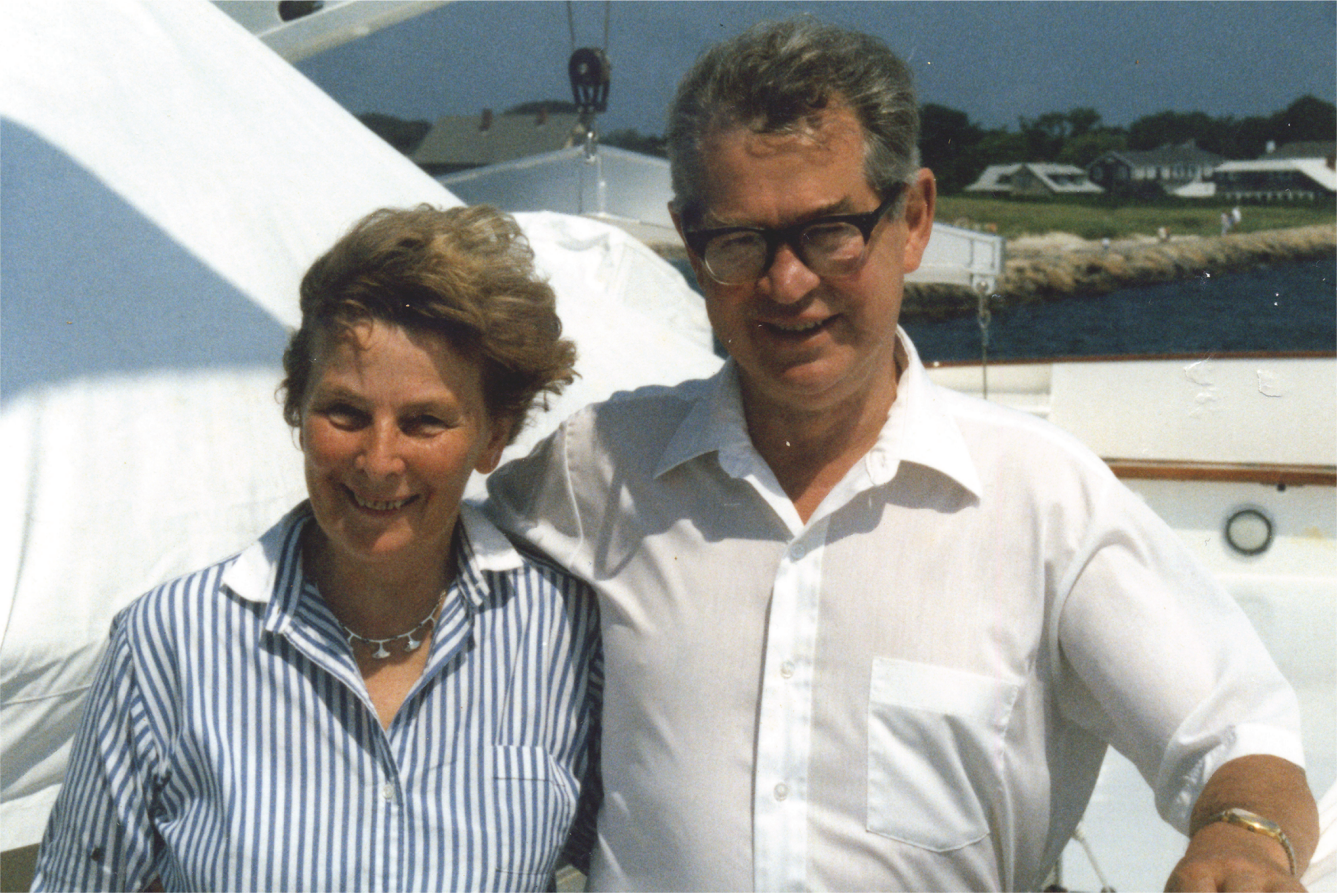
John with his wife, Evelyn, on a sail boat in Woods Hole, circa August 1986.

Steele’s first degree was in pure math, “with the emphasis on *pure*”, “…the highlight of my time [during his degree] was the announcement of a simple and elegant proof of the prime number theorem, a result already proven but the previous proof had been considered aesthetically unsatisfactory” (Steele, 1997). He would likely have appreciated the elegance of the proofs but was nevertheless most impressed by practical application of math to real world problems. Furthermore, math had to be meaningful for everyone in the scientific community, and Steele was a genius when it came to effective communication with non-modellers. Several factors contributed to this success. First, he employed a straightforward experiment-like approach to applying his models, what he called “hypothesis-test-hypothesis” (Steele, 1977), in which parameter values were varied one at a time to demonstrate the roles of individual factors in controlling system dynamics. Second, Steele ensured that the simplifications and assumptions in the models were described in scrupulous detail and the resulting analyses were framed in terms of simple, logical conjectures. Most of all, Steele “just loved participating with, and interacting with, other people concerning the advancement of knowledge” (Gagosian, 2014) and, in particular, “sought out interactions with younger scientists, which was a hallmark of his career” (Hofmann, 2014). He developed many successful collaborations throughout his career. An amusing example took place in 1974 when Bruce Frost, an expert on zooplankton feeding behaviour (e.g. Frost, 1972), went on vacation to La Jolla, California, with his wife and children. He started reading a copy of Steele’s newly published book on marine ecosystems (Steele, 1974) as the family left Seattle and was so captivated by Steele’s use of modelling that he had to keep reading it, cover to cover, leaving his wife to drive the whole way to California (Gentleman, 2002)! Frost subsequently spent a sabbatical with Steele working on size-dependent relations in plankton communities (Steele and Frost, 1977) and went on to undertake further modelling studies, publishing key papers on the role of grazing and iron in maintaining HNLC systems (Frost, 1987, 1991; Frost and Franzen, 1992).

Models should always be developed in context of the real world. Tuning to, and validation against, data are the preoccupation of many a modeller. Indeed, one can take the view that modellers spend too much time tuning parameters in order to ensure any desired outcome (Aber, 1997), a bit like turning the dials on a toaster until the toast comes out just right (Franks, 2009). Data are, of course, important, especially for validation in case of models used to make predictions such as the future state of the ocean under anthropogenic forcing. Steele himself never lost sight of the ultimate goal of developing models to inform policy and management, especially fisheries (e.g. Steele *et al.*, 2013). He was a fan of modern end-to-end models, emphasizing the importance of both physical context (Ruzicka *et al.*, 2018) and data independence: “The level of insight gained and the usefulness of the scenarios will be a function of the degree of independence of the data used to drive the simulation, from the data used to assess the output” (Steele, 2012).

It is, however, remarkable that no data are shown in SH92, where the emphasis was on gaining knowledge and understanding, rather than prediction. The seasonal cycles of plankton and nutrients are shown qualitatively for the two sites, based on observations. And Steele gathered as much information as he could about the organisms involved, as well as on processes such as growth, grazing and mortality. When it came to analysing and interpreting model results, Steele’s main aim was, remarkably, to look for qualitative agreement in order to explore ideas and concepts, without the need for precise fitting to data. His ideas were subject to evaluation by hypothesis-test-hypothesis, as in the classic philosophical view of hypotheses that act as “nets cast to catch what we call ‘the world’: to rationalise, to explain, and to master it” (Popper, 1959). In the event that a credible qualitative agreement was obtained, the model could be used to estimate unmeasured quantities and assess the relative importance (model sensitivity) to different aspects of the system. If the fit was not so good, the model could be used to identify gaps in knowledge: “By forcing one to produce formulas to define each process and put numbers to the coefficients, [a simulation of a natural ecosystem] reveals the lacunae in one’s knowledge...the main aim is to determine where the model breaks down and use it to suggest further field or experimental work” (Steele, 1974). Scientific method at its best.

To conclude, we wish to pay a final tribute to John. He was inspirational and accomplished as a scientist. Yet he was not only the erudite professional but also the consummate gentleman. He and his wife Evelyn (to whom he was married for 57 years; Fig. 6) hosted many gatherings of scientists, both in their cottage in Scotland and their home in Cape Cod. “He was good company”, she said, and a great conversationalist (Lawrence, 2013). Steele was visionary in his thinking, decades ahead of his time, and his papers remain classics in the field, as relevant today as when they were published. As John Cullen put it, “Steele was indeed a giant in our field… he established the foundations of modern oceanographic research” and yet “shy and humble, he seemed uncomfortable taking credit for his brilliant work” (Lawrence, 2013). Science was an adventure for Steele, a journey of discovery, but which always maintained focus on practical application to societal issues of modern times. “His twinkle is gone, but his brilliant star shines on: thank you, John Steele” Gentleman (2014).

## FUNDING

National Capability funding from the Natural Environment Research Council, UK, programme CLASS (NE/R015953/1 to T.R.A.); Natural Sciences and Engineering Research Council of Canada (W.C.G.).
